# Thyroid autoantibodies predict long-term treatment response in euthyroid chronic spontaneous urticaria: a retrospective cohort study with propensity score matching

**DOI:** 10.3389/fmed.2026.1782727

**Published:** 2026-03-23

**Authors:** Lizhen Zhuang, Caihua Zhuang, Huixin Jiang, Shanshan Su

**Affiliations:** Department of Dermatology, Dehua County Hospital, Quanzhou, Fujian, China

**Keywords:** biomarker, chronic spontaneous urticaria, euthyroid, thyroid autoantibodies, treatment response

## Abstract

**Background:**

Chronic spontaneous urticaria (CSU) affects 0.5–1% of the population, with 40% remaining refractory to guideline-recommended therapy. Thyroid autoantibodies (TPOAb/TgAb) coexist in 20–30% of cases, yet their prognostic value in euthyroid patients remains controversial.

**Methods:**

We conducted a propensity score-matched retrospective cohort study of 350 euthyroid CSU patients between 2020 and 2023. The primary endpoint was Week 24 treatment response (complete/partial response defined by UAS7/UCT scores). Secondary outcomes included time-to-response, relapse rates, and treatment escalation requirements. Conditional logistic regression and Cox proportional hazards models assessed associations.

**Results:**

Antibody-positive patients exhibited significantly lower Week 24 response rates (47.6% vs. 69.4%; *p* < 0.001). Median time-to-response was delayed by 4.0 weeks (14.0 vs. 10.0 weeks, HR 0.56, *p* < 0.001). Relapse rates were higher among responders (27.1% vs. 14.0%, *p* = 0.036), and treatment escalation was more frequent (omalizumab: 38.7% vs. 21.0%; cyclosporine: 12.9% vs. 4.8%, both *p* < 0.001). A dose–response gradient was observed, with dual-positive patients showing the poorest outcomes. Effects were amplified in severe disease (UAS7 ≥ 16) and omalizumab-treated subgroups.

**Conclusion:**

Thyroid autoantibody positivity independently predicts inferior long-term treatment response, delayed therapeutic onset, and increased relapse risk in euthyroid CSU patients. Routine TPOAb/TgAb screening at diagnosis could enable personalized treatment escalation, improving outcomes.

## 1 Introduction

Chronic spontaneous urticaria (CSU) is a debilitating skin disease characterized by recurrent, unpredictable wheals and/or angioedema persisting for ≥6 weeks, affecting approximately 0.5–1% of the global population with substantial impairment of quality of life (1, 2). Despite recent advances in understanding its pathogenesis, nearly 50% of patients remain refractory to guideline-recommended high-dose H1-antihistamines, necessitating escalation to expensive biologics such as omalizumab or immunosuppressants like cyclosporine (3, 4). The absence of reliable predictors for treatment response compels clinicians to adopt a trial-and-error approach, prolonging disease burden and increasing healthcare costs.

Emerging evidence indicates that autoimmune mechanisms are involved in up to 50% of CSU cases, where functional autoantibodies against IgE or its high-affinity receptor (FcεRI) trigger mast cell activation (5, 6). Notably, thyroid autoimmunity frequently coexists with CSU, with 30–40% of patients exhibiting elevated anti-thyroid peroxidase (TPOAb) and/or anti-thyroglobulin antibodies (TgAb) compared to <10% in the general population (7–9). Anti-thyroid peroxidase and anti-thyroglobulin autoantibodies of the IgG class do not release histamine per se, but are often associated with the occurrence of histamine-releasing anti-FcεRI and anti-IgE autoantibodies (10, 11). While IgG autoantibodies directed against high-affinity IgE receptor or IgE trigger histamine release from mast cells, thyroid autoantibodies themselves serve as serological markers of this underlying autoimmune mechanism rather than direct effectors. Notably, anti-thyroid IgE autoantibodies may contribute directly to mast cell activation, but these are not measured in routine clinical practice and were not evaluated in this study. The clinical implications of thyroid autoantibody positivity still remain a subject of controversy, as there are conflicting reports regarding their association with disease severity, treatment resistance, and long-term prognosis (12–14).

Most prior investigations have been limited by cross-sectional designs, small sample sizes, and short follow-up durations, failing to establish whether thyroid autoantibodies truly predict therapeutic outcomes rather than merely reflecting disease activity (15–18). Critically, few studies have exclusively examined euthyroid patients, raising concerns that subclinical thyroid dysfunction may confound associations.

We hypothesized that thyroid autoantibody positivity independently predicts inferior long-term treatment response in euthyroid CSU patients, with antibody titer correlating linearly with treatment failure risk. To test this, we conducted a propensity score-matched retrospective cohort study with 24-week follow-up, restricting our analysis to euthyroid individuals to isolate the immunological effects of autoantibodies from thyroid hormone-mediated pathways. By leveraging a large, well-characterized clinical database and rigorous confounding control, this study aims to fill a critical evidence gap and provide actionable biomarkers for personalized CSU management.

## 2 Methods

### 2.1 Study design and setting

This single-center retrospective cohort study was conducted with propensity score matching (PSM) at our hospital between January 2020 and December 2023. The study protocol received approval from the Institutional Review Board (IRB No. 2019[006]) and adhered to the Declaration of Helsinki principles. Given its retrospective nature, the requirement for informed consent was waived. We followed the STROBE guidelines for cohort studies and the RECORD statement for observational research utilizing routinely collected health data.

### 2.2 Study participants

Patients were retrospectively identified through electronic medical record queries using ICD-10 codes for CSU. Eligible participants were adults aged 18 years or older with a confirmed CSU diagnosis according to the EAACI/GA^2^LEN/EDF/WAO 2021 consensus guidelines, defined by recurrent wheals and/or angioedema persisting for more than 6 weeks without identifiable external triggers (2). All included patients had documented euthyroid status at baseline, with thyroid-stimulating hormone levels between 0.5 and 4.0 mIU/L and free thyroxine within the institutional reference range. Complete measurements of IgG-class thyroid peroxidase (TPOAb) and thyroglobulin (TgAb) autoantibodies at diagnosis were mandatory, as was availability of baseline Urticaria Activity Score 7 (UAS7) and a minimum of 24 weeks of follow-up data. Patients were excluded if they had any prior history of thyroid disease, including Hashimoto’s thyroiditis or Graves’ disease, or had undergone thyroid surgery. Those with subclinical hypothyroidism (TSH > 4.0 mIU/L) or hyperthyroidism (TSH < 0.5 mIU/L), positive thyroid-stimulating hormone receptor antibodies, or who were pregnant or within 12 months postpartum were also excluded. Additionally, individuals with coexisting autoimmune diseases such as systemic lupus erythematosus, rheumatoid arthritis, or type 1 diabetes, those with chronic inducible or mixed urticaria, active malignancy, or those receiving immunosuppressive therapy, and patients with incomplete treatment response documentation or follow-up duration less than 24 weeks were omitted from the analysis.

### 2.3 Exposure definition

The primary exposure was thyroid autoantibody status, dichotomized based on established laboratory thresholds. Patients with anti-thyroid peroxidase antibody levels exceeding 34 IU/mL and/or anti-thyroglobulin antibody levels above 115 IU/mL, as quantified by electrochemiluminescence immunoassay (Cobas e601, Roche Diagnostics), were classified as antibody-positive. Those with both TPOAb ≤34 IU/mL and TgAb ≤115 IU/mL constituted the antibody-negative reference group. For secondary analyses, participants were further stratified into three mutually exclusive categories: isolated TPOAb positivity, isolated TgAb positivity, and dual antibody positivity.

### 2.4 Outcome measures

The primary outcome was long-term treatment response assessed at Week 24 using a composite endpoint. Complete response was defined as achievement of UAS7 = 0 together with a Urticaria Control Test score ≥13 maintained for at least four consecutive weeks. UAS7 scores were assessed by trained dermatologists with inter-rater reliability *κ* = 0.85 (95% CI: 0.78–0.92) in 30 random cases. Partial response was characterized by a ≥ 50% reduction for ≥4 consecutive weeks in UAS7 from baseline. Non-response included patients with <50% reduction in baseline UAS7 or those experiencing disease worsening. The overall response rate was calculated as the proportion achieving either complete or partial response. Secondary outcomes included time to response, measured as weeks from treatment initiation to the first documented partial or complete response; relapse rate among those achieving complete response who subsequently developed UAS7 ≥ 6 during weeks 12–24; angioedema resolution in patients presenting with baseline angioedema; and the proportion requiring treatment escalation to omalizumab or cyclosporine due to antihistamine resistance.

### 2.5 Data collection

Trained investigators extracted data from electronic health records using a standardized electronic case report form. Baseline demographics included age, sex, body mass index, disease duration, and smoking status. Clinical parameters comprised baseline UAS7, Urticaria Control Test scores, presence and severity of angioedema, prior treatment history, and total IgE levels. Laboratory data encompassed thyroid function tests (TSH), thyroid autoantibody titers, and inflammatory markers including C-reactive protein. Comorbid allergic conditions such as atopic dermatitis, allergic rhinitis, and asthma were documented. Information on treatment regimens, including second-generation antihistamine type, dose escalation patterns, omalizumab administration, and cyclosporine use, was recorded for all participants. Longitudinal follow-up data were captured at weeks 4, 12 and 24 through outpatient clinic visits or structured telemedicine consultations, with UAS7 scores documented at each time point.

### 2.6 Propensity score matching

To mitigate selection bias arising from the non-random distribution of thyroid autoantibodies, we employed propensity score matching to create comparable cohorts. Propensity scores were estimated using logistic regression modeling the probability of being antibody-positive as a function of clinically relevant baseline covariates, including age, sex, body mass index, disease duration, baseline UAS7, log-transformed total IgE, presence of angioedema, and prior antihistamine use. We implemented 1:1 nearest-neighbor matching without replacement, applying a caliper width of 0.2 standard deviations of the logit-transformed propensity score to ensure adequate matches while minimizing residual confounding. Covariate balance between matched groups was assessed using standardized mean differences, with values less than 0.10 indicating acceptable balance. When initial matching failed to achieve balance, we iteratively refined the matching algorithm by adjusting the caliper width and matching ratio until all predefined covariates achieved satisfactory balance.

### 2.7 Statistical analysis

Continuous variables were expressed as mean with standard deviation or median with interquartile range based on normality assessment using the Shapiro–Wilk test, while categorical variables were presented as frequencies and percentages. The primary analysis compared Week 24 response rates between antibody-positive and antibody-negative groups in the propensity-matched cohort using McNemar’s test for paired binary data, with conditional logistic regression employed to estimate odds ratios (OR) and 95% confidence intervals (CI). Secondary analyses utilized Kaplan–Meier survival curves and Cox proportional hazards regression with robust standard errors to evaluate time to response, while accounting for the matched nature of the data. Subgroup analyses examined treatment response stratified by antibody type, baseline disease severity, treatment modality and age. We explored potential non-linear relationships between continuous antibody titers and treatment outcomes using restricted cubic splines. Sensitivity analyses were conducted to assess robustness, including multiple imputation by chained equations for missing UAS7 data that remained below 5%, inverse probability weighting as an alternative to propensity score matching, instrumental variable analysis using hospital admission year as a plausible instrument, and calculation of E-values to quantify the potential impact of unmeasured confounding. All statistical analyses were performed using R version 4.3.0 with the MatchIt and survival packages, with a two-sided *p*-value threshold of 0.05 defining statistical significance.

Power calculations were based on pilot data showing 24-week response rates of 48% in antibody-positive versus 71% in antibody-negative euthyroid CSU patients. To detect this 23% absolute difference with 80% power at a two-sided alpha level of 0.05, we required 105 matched pairs. Anticipating a 15% reduction in effective sample size due to potential match loss and attrition, we aimed to include an initial cohort of at least 350 euthyroid CSU patients to ensure adequate statistical power for the primary endpoint while preserving precision for secondary analyses.

## 3 Results

### 3.1 Study population and baseline characteristics

**Figure 1:**
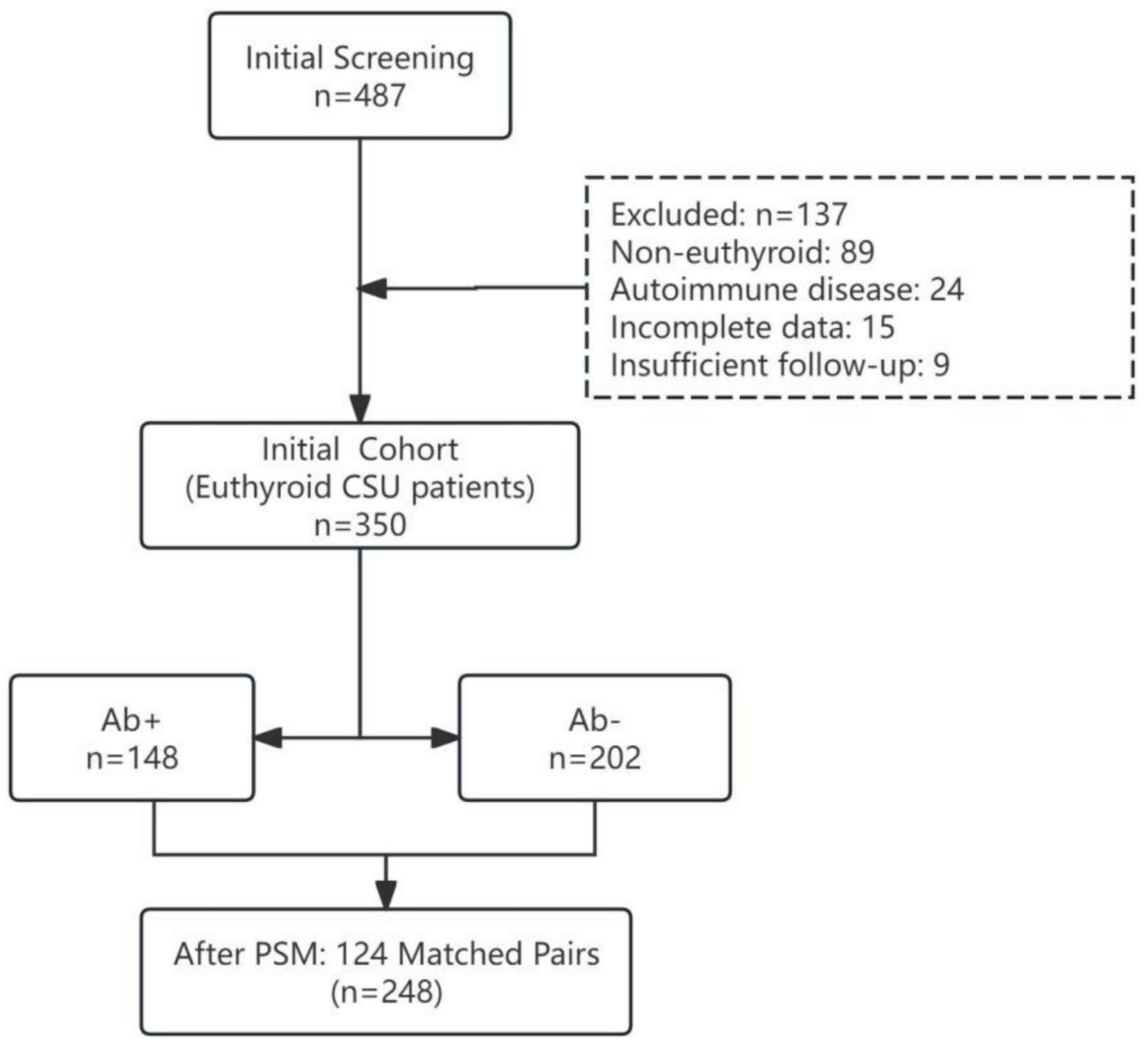
Study flow diagram.

Between January 2020 and December 2023, 487 patients with CSU were screened for eligibility. Of these, 89 were excluded for non-euthyroid status, 24 for coexisting autoimmune disease, 15 for incomplete thyroid antibody data, and 9 for insufficient follow-up duration, leaving 350 euthyroid CSU patients in the initial cohort (Figure 1).

The prevalence of thyroid autoantibody positivity was 42.3% (148/350). Prior to propensity score matching, antibody-positive patients exhibited higher baseline UAS7 scores (median 18 [IQR 15–22] vs. 14 (11–18), *p* < 0.001) and longer disease duration (mean 14.2 ± 8.7 months vs. 10.5 ± 6.9 months, *p* < 0.001) compared to antibody-negative patients. After 1:1 nearest-neighbor matching, 124 well-balanced pairs were created with standardized mean differences <0.10 for all covariates (Supplementary Figure 1). The final matched cohort (*n* = 248) had a mean age of 38.6 ± 12.3 years, 64.5% were female, and median baseline UAS7 was 16 (IQR 13–20) in both groups (Table 1). Within the antibody-positive arm, 58 pairs had isolated TPOAb positivity, 28 pairs isolated TgAb positivity, and 38 pairs dual positivity.

**Table 1:** Baseline characteristics of euthyroid CSU patients.

Characteristic	Overall cohort (*n* = 350)	Pre-PSM			Post-PSM	
		Ab+ (*n* = 148)	Ab− (*n* = 202)	*p*	Ab+ (*n* = 124)	Ab− (*n* = 124)
Demographics						
Age, years	38.4 ± 12.1	39.2 ± 12.5	37.8 ± 11.8	0.28	38.9 ± 12.6	38.3 ± 11.9
Female sex, *n* (%)	226 (64.6)	98 (66.2)	128 (63.4)	0.58	81 (65.3)	79 (63.7)
BMI, kg/m^2^	24.8 ± 4.2	25.1 ± 4.5	24.6 ± 4.0	0.31	24.9 ± 4.3	24.7 ± 4.1
Disease features						
Disease duration, months	12.1 ± 7.9	14.2 ± 8.7	10.5 ± 6.9	<0.001	12.8 ± 8.1	12.5 ± 7.8
Baseline UAS7, median (IQR)	16 (13–20)	18 (15–22)	14 (11–18)	<0.001	16 (13–20)	16 (13–20)
Angioedema, *n* (%)	128 (36.6)	64 (43.2)	64 (31.7)	0.02	52 (41.9)	50 (40.3)
Prior antihistamine use, *n* (%)	210 (60.0)	98 (66.2)	112 (55.4)	0.03	76 (61.3)	74 (59.7)
Laboratory parameters						
TSH, mIU/L	2.1 ± 0.9	2.2 ± 0.8	2.0 ± 0.9	0.12	2.1 ± 0.8	2.0 ± 0.9
Total IgE, kU/L, median (IQR)	85 (42–156)	92 (48–178)	78 (38–142)	0.08	88 (45–162)	86 (43–158)
TPOAb titer, IU/mL, median (IQR)	—	156 (68–432)	—		148 (65–398)	—
TgAb titer, IU/mL, median (IQR)	—	245 (89–567)	—		238 (85–545)	—
CRP, mg/L	3.2 ± 2.8	3.8 ± 3.1	2.8 ± 2.5	0.03	3.5 ± 3.0	3.3 ± 2.9

Ab+, antibody-positive; Ab−, antibody-negative; PSM, propensity score matching; IQR, interquartile range; UAS7, Urticaria Activity Score 7-day; CRP, C-reactive protein.

### 3.2 Association between thyroid autoantibodies and treatment response

Antibody-positive patients demonstrated significantly lower overall response rates at Week 24 (47.6% vs. 69.4%; conditional OR 0.38, 95% CI 0.23–0.62, *p* < 0.001). Complete response rates were similarly reduced (22.6% vs. 41.9%, *p* = 0.002), yielding an absolute risk difference of 21.8% (95% CI 10.3–33.3%) and a number needed to harm of 4.6 (Table 2).

**Table 2:** Association between thyroid autoantibodies and treatment response.

Outcome	Ab+ (*n* = 124)	Ab− (*n* = 124)	Conditional OR (95% CI)	*p*-value
Response category				
Complete response, *n* (%)	28 (22.6)	52 (41.9)	0.39 (0.23–0.68)	0.002
Partial response, *n* (%)	31 (25.0)	34 (27.4)	0.89 (0.51–1.54)	0.67
Non-response, *n* (%)	65 (52.4)	38 (30.6)	0.38 (0.23–0.62)	<0.001
Overall response rate^†^, *n* (%)	59 (47.6)	86 (69.4)	0.38 (0.23–0.62)	<0.001
Absolute risk difference	-	-	−21.8% (−33.3 to −10.3)	<0.001
Number needed to harm	-	-	4.6(95% CI 3–11)	-

Conditional OR from conditional logistic regression adjusting for matched pairs.

^†^Complete + partial response.

The association remained robust after adjusting for residual IgE imbalance (adjusted OR 0.41, 95% CI 0.25–0.68). A clear dose–response gradient emerged: dual-positive patients showed the lowest response rate (36.8%), followed by isolated TPOAb (51.7%) and TgAb positivity (53.6%), compared to 69.4% in the antibody-negative group (*p* for trend <0.001).

### 3.3 Response kinetics and increased relapse risk

**Figure 2:**
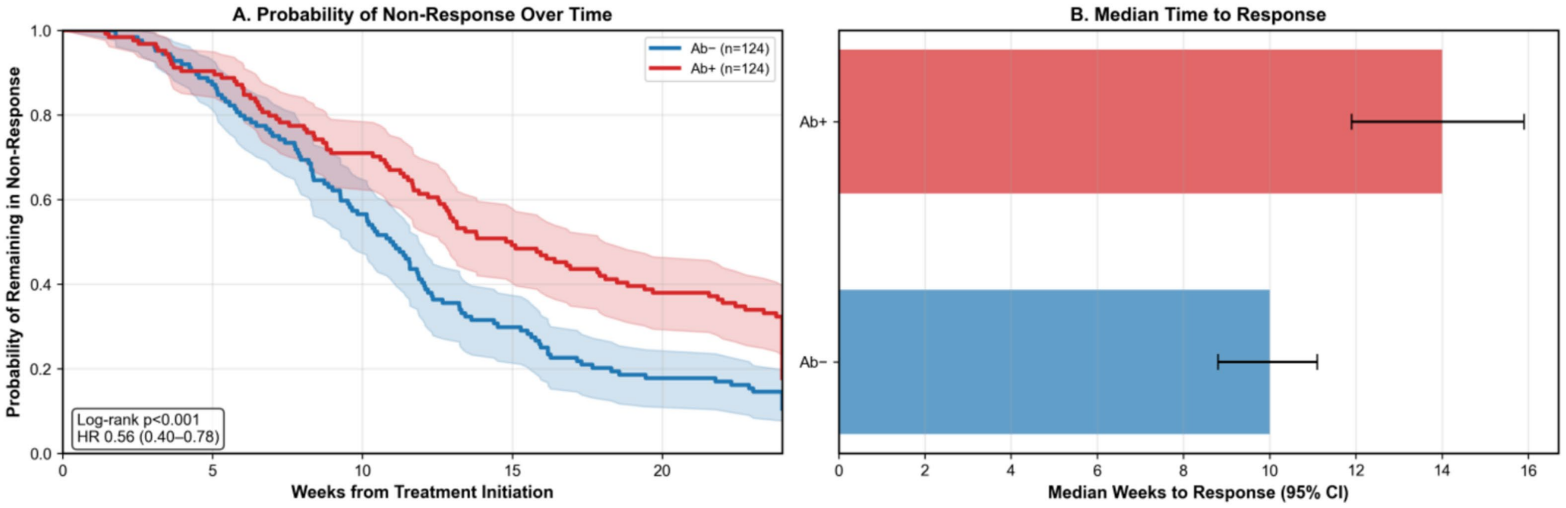
Kaplan–Meier curve for time to treatment response. **(A)** Probability of achieving partial or complete response over 24 weeks in propensity-matched cohort. **(B)** Median time to response was 14.0 weeks (95% CI 12.1–15.9) in antibody-positive patients vs. 10.0 weeks (95% CI: 8.8–11.2) in antibody-negative patients (log-rank *p* < 0.001). Hazard ratio 0.56 (95% CI: 0.40–0.78). Shaded areas represent 95% confidence bands. Number at risk shown below each time point.

Time-to-response analysis revealed antibody-positive patients required a median of 14.0 weeks (95% CI 12.1–15.9) versus 10.0 weeks (95% CI 8.8–11.2) in antibody-negative patients (log-rank *p* < 0.001). Cox regression confirmed the association (adjusted HR 0.56, 95% CI: 0.40–0.78) (Figure **2**).

Among patients achieving complete response, relapse rates during weeks 12–24 were higher in the antibody-positive group (27.1% [16/59] vs. 14.0% [12/86], conditional OR 2.32, 95% CI 1.06–5.08, *p* = 0.036). Angioedema resolution was also inferior (58.3% vs. 78.9%, *p* = 0.018). Treatment escalation was more frequent in antibody-positive patients, with 38.7% requiring omalizumab and 12.9% cyclosporine versus 21.0 and 4.8%, respectively, in antibody-negative patients (*p* < 0.001) (Table 3).

**Table 3:** Response kinetics and increased relapse risk.

Outcome	Ab+ (*n* = 124)	Ab− (*n* = 124)	Effect estimate (95% CI)	*p*-value
Time to response (weeks)	14.0 (12.1–15.9)	10.0 (8.8–11.2)	HR 0.56 (0.40–0.78)	<0.001
Relapse rate, *n* (%)	16/59 (27.1)	12/86 (14.0)	conditional OR 2.32 (1.06–5.08)*	0.036
Angioedema resolution, *n* (%)	27/52 (58.3)	39/50 (78.9)	conditional OR 0.38 (0.16–0.89)^†^	0.018
Treatment escalation				
Omalizumab use, *n* (%)	48 (38.7)	26 (21.0)	conditional OR 2.41 (1.39–4.17)	<0.001
Cyclosporine use, *n* (%)	16 (12.9)	6 (4.8)	conditional OR 2.89 (1.07–7.81)	0.037

^*^Among patients achieving complete response.

^†^Among patients with baseline angioedema.

### 3.4 Pronounced antibody effects in severe disease and omalizumab cohort

Subgroup analyses demonstrated that the deleterious effect of autoantibodies was amplified in patients with severe baseline disease (UAS7 ≥ 16: 39.3% vs. 63.8% response, conditional OR 0.32, 95% CI 0.16–0.64) compared to mild–moderate disease (*p* for interaction = 0.048). The association was particularly striking in omalizumab-treated patients, where antibody positivity conferred a 54.2% response rate versus 81.5% in antibody-negative patients (conditional OR 0.28, 95% CI 0.12–0.66, *p* = 0.003) (Table 4).

**Table 4:** Subgroup analyses of week 24 response rate.

Subgroup	Ab+ response rate	Ab− response rate	Conditional OR (95% CI)	*p* for interaction
Baseline severity				0.048
UAS7 < 16 (mild/moderate)	58.1% (25/43)	75.6% (34/45)	0.48 (0.24–0.98)	
UAS7 ≥ 16 (severe)	39.3% (35/89)	63.8% (57/89)	0.32 (0.16–0.64)	
Antibody type				0.19
TPOAb+ only	51.7% (30/58)	69.4% (86/124)	0.46 (0.25–0.84)	
TgAb+ only	53.6% (15/28)	69.4% (86/124)	0.51 (0.23–1.13)	
Dual positive	36.8% (14/38)	69.4% (86/124)	0.25 (0.12–0.53)	
Treatment modality				0.003
Antihistamine only	55.3% (26/47)	73.5% (50/68)	0.45 (0.22–0.92)	
Omalizumab treated	54.2% (26/48)	81.5% (22/27)	0.28 (0.12–0.66)	
Age group				0.52
<40 years	46.8% (37/79)	70.8% (46/65)	0.36 (0.19–0.69)	
≥40 years	48.9% (22/45)	67.8% (40/59)	0.41 (0.20–0.85)	

**Figure 3:**
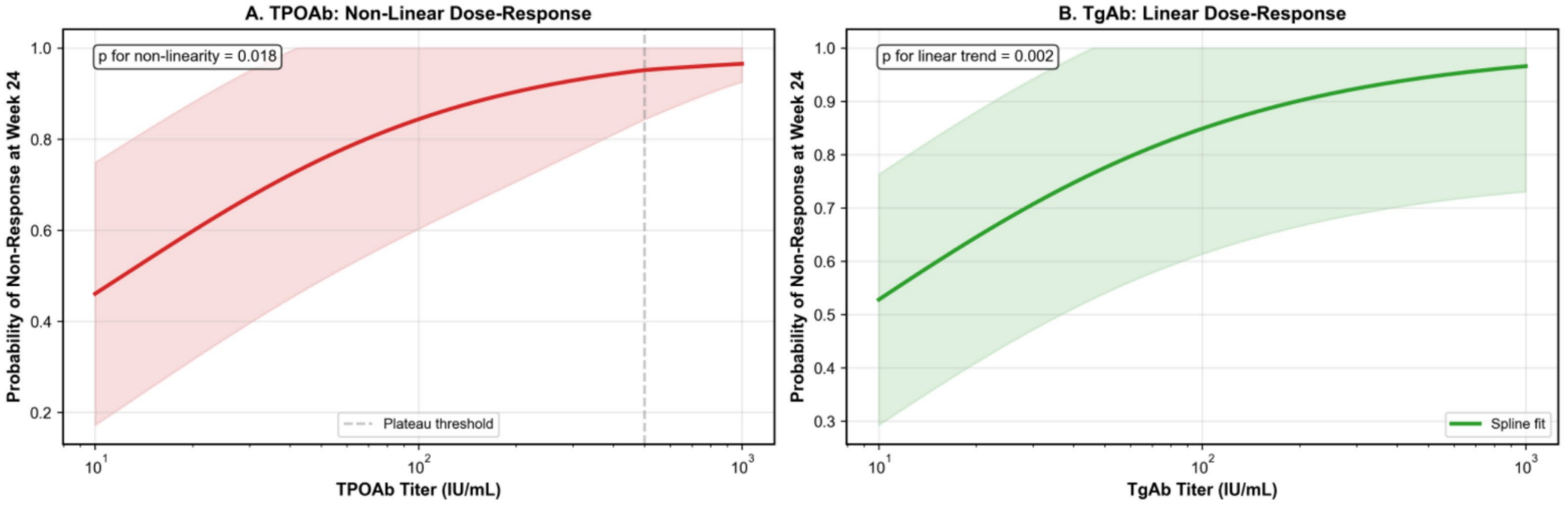
Restricted cubic spline analysis. **(A)** Relationship between continuous TPOAb titer and probability of non-response at week 24, adjusted for baseline covariates. The spline curve with four knots (at 50, 100, 400, and 1,000 IU/mL) demonstrates a non-linear association (*p* for non-linearity = 0.018), with risk plateauing above 500 IU/mL. Reference set at 0 IU/mL. Gray shading indicates 95% confidence interval. **(B)** Linear relationship between log-transformed TgAb titer and non-response probability (*p* = 0.002 for linear trend). Each doubling of TgAb titer was associated with 12% increased odds of non-response (OR 1.12, 95% CI: 1.04–1.20).

Restricted cubic spline analysis revealed a non-linear relationship between TPOAb titer and non-response risk, plateauing above 500 IU/mL (*p* for non-linearity = 0.018), whereas TgAb showed a linear dose–response (*p* = 0.002) (Figure 3).

### 3.5 Sensitivity analyses

Findings were robust across multiple sensitivity analyses. Multiple imputation for 4.4% of patients with missing Week 24 data yielded an identical OR of 0.37 (95% CI 0.22–0.61). Inverse probability weighting (OR 0.40, 95% CI 0.24–0.67) and instrumental variable analysis (causal risk difference −0.19, 95% CI − 0.34 to −0.04) produced consistent estimates. The E-value of 3.2 suggested strong resilience against unmeasured confounding. Excluding patients with borderline TSH (3.5–4.0 mIU/L) did not alter conclusions (OR 0.36, 95% CI 0.21–0.61). Antibody tertile analysis confirmed a dose–response relationship, with the highest tertile showing 2.7-fold increased odds of non-response (*p* for trend <0.001) (Supplementary Figure 2).

## 4 Discussion

This propensity score-matched retrospective cohort study demonstrates that thyroid autoantibody positivity independently predicts inferior long-term treatment response, delayed therapeutic onset, and increased relapse risk in euthyroid CSU patients. At Week 24, antibody-positive patients exhibited a 21.8% absolute reduction in response rates (47.6% vs. 69.4%, conditional OR 0.38, 95% CI 0.23–0.62) and required a median of 4 additional weeks to achieve response compared to antibody-negative counterparts. Notably, the association followed a clear dose–response gradient, with dual-positive patients showing the poorest outcomes (36.8% response rate). These findings persisted across multiple sensitivity analyses and were most pronounced in severe disease and omalizumab-treated subgroups, highlighting the clinical utility of TPOAb and TgAb as prognostic biomarkers for therapeutic stratification.

Our results corroborate and extend prior investigations linking thyroid autoimmunity with CSU refractoriness. Jang et al. reported antihistamine resistance in 46% of CSU patients with thyroid autoantibodies, and Alsaffar et al. observed a higher need for omalizumab in children with elevated anti-TPO levels (16, 19, 20). In an 8-week follow-up study of patients with severe chronic urticaria receiving omalizumab treatment, TPOAb were found to be negatively correlated with a favorable clinical response (21). By implementing 24-week follow-up and propensity score matching, our study overcomes these limitations, providing the robust evidence that autoantibody status predicts durable therapeutic response rather than transient disease activity. However, conflicting results from prior studies require clarification. For instance, a retrospective analysis of refractory severe CSU patients treated with omalizumab found no correlation between autoimmune thyroid disease history and treatment response (22), while other studies reported thyroid autoimmunity cannot serve as a predictor of omalizumab response or Type I/IIb CSU marker (23, 24). These discrepancies likely stem from three key factors: inclusion of patients with pre-existing thyroid disease or subclinical hypothyroidism (TSH > 4.0 mIU/L), where thyroid hormone fluctuations may confound immunological effects; heterogeneity in patient disease severity and follow-up durations; and inadequate sample sizes in smaller studies, limiting power to detect modest effect sizes. In contrast, our strict euthyroid inclusion criteria (TSH 0.5–4.0 mIU/L) isolate autoimmune effects from endocrine confounding, and our matched cohort of 248 patients provides >80% power to detect a 20% absolute difference—addressing critical limitations of prior work.

The dose–response relationship we observed aligns with emerging mechanistic insights into CSU pathogenesis. Higher autoantibody titers correlate with greater basophil activation via anti-FcεRI IgG and enhanced complement C5a-mediated mast cell degranulation (25). Dual-positive patients exhibited the highest non-response rates, suggesting synergistic pathogenicity consistent with reports that combined TPOAb/TgAb positivity amplifies Th17-driven inflammation in autoimmune diseases. Several mechanisms may explain the adverse impact of thyroid autoantibodies on therapeutic response. First, TPOAb and TgAb are not able to activate mast cells and basophils per se. Instead, these thyroid autoantibodies serve as robust markers of autoimmune diathesis that are often associated with the presence of IgG autoantibodies directed against FcεRI or IgE—the established effectors capable of cross-linking FcεRI on mast cells and basophils, thereby bypassing the IgE-mediated pathway targeted by antihistamines and omalizumab (26). It is this co-occurrence with functional autoantibodies, rather than direct action of TPOAb/TgAb, that likely explains the attenuated efficacy of high-dose antihistamines and omalizumab in our antibody-positive cohort. Second, so far, it has not been demonstrated that TPOAb and TgAb can induce histamine release from mast cells and basophils following complement fixation and generation of C5a. While complement activation may contribute to CSU pathogenesis through C5a-mediated effects, this mechanism has not been specifically linked to thyroid autoantibodies themselves (27). The delayed time-to-response in antibody-positive patients may reflect slower suppression of this complement-mediated pathway. Third, thyroid autoimmunity is characterized by Th1-dominant responses and elevated IL-17, cytokines implicated in CSU refractoriness (28). Our dose–response findings support a model where progressive loss of immune tolerance, mirrored by rising antibody titers, drives disease persistence through IL-17-mediated neutrophil recruitment and keratinocyte activation (29). Finally, emerging data implicate IL-24 as a key effector in autoantibody-positive CSU, with levels correlating with UAS7 and predicting omalizumab resistance (30). Thyroid autoantibodies may induce IL-24 production from Th17 cells, creating a feed-forward loop that sustains urticaria despite therapy. Future studies integrating these mechanistic biomarkers are warranted to validate causal pathways.

Our findings have immediate translational relevance. First, routine TPOAb/TgAb measurement at CSU diagnosis could identify the ~40% of patients at high risk for treatment failure, enabling earlier escalation to omalizumab or adjunctive therapies (e.g., cyclosporine, dupilumab) rather than prolonged antihistamine trials. The absolute risk difference of 21.8% suggests a number needed to screen of 5 to prevent one treatment failure through early intervention, improving cost-effectiveness. Second, antibody titer monitoring may guide therapy intensity. Our restricted cubic spline analysis indicates that TPOAb >500 IU/mL exhibits maximal risk plateau, suggesting a potential therapeutic threshold for aggressive management. The linear TgAb relationship implies that even modest elevations predict incremental risk, supporting universal screening rather than selective testing. Third, the higher relapse rate among antibody-positive responders (27.1% vs. 14.0%) indicates that antibody positivity identifies a subgroup requiring extended maintenance therapy rather than early discontinuation.

Several limitations merit acknowledgment. First, the retrospective design cannot establish causality, and residual confounding from unmeasured variables (e.g., vitamin D status, psychological stress) remains possible. The calculated E-value of 3.2 indicates moderate resilience against unmeasured confounding, but a strong confounder could still explain our findings. Second, single-center data from an Asian population may limit generalizability to other ethnicities with different baseline autoimmunity prevalence. Multi-center validation is needed. Third, we did not measure functional autoantibodies (anti-FcεRI IgG), IL-24, or Th17/Treg ratios, precluding mechanistic confirmation. Fourth, treatment adherence was assumed complete based on prescription records, though real-world compliance may vary and could differentially affect outcomes if antibody-positive patients exhibit poorer adherence due to psychological comorbidities.

## 5 Conclusion

This propensity score-matched retrospective cohort study provides robust evidence that thyroid autoantibody positivity independently predicts poor long-term treatment response, delayed time-to-response, and increased relapse risk in euthyroid CSU patients. The dose–response relationship and pronounced effects in severe disease and omalizumab-treated subgroups highlight the clinical utility of TPOAb/TgAb as prognostic biomarkers for therapeutic stratification. These findings advocate for routine thyroid autoantibody screening at CSU diagnosis to identify high-risk patients who may benefit from early escalation therapy, potentially transforming the current one-size-fits-all treatment paradigm into a precision medicine approach. Prospective validation and mechanistic studies are warranted to translate these observations into evidence-based guidelines.

## Data Availability

The original contributions presented in the study are included in the article/Supplementary material, further inquiries can be directed to the corresponding author.
