# Supplementary material for: Thyroid autoantibodies predict long-term treatment response in euthyroid chronic spontaneous urticaria: a retrospective cohort study with propensity score matching
Source: Front Med (Lausanne). 2026 Mar 23;13:1782727. doi: 10.3389/fmed.2026.1782727 (PMC13050742; doi:10.3389/fmed.2026.1782727)
Supplement: Supplementary file 1 [file Supplementary_file_1.docx]

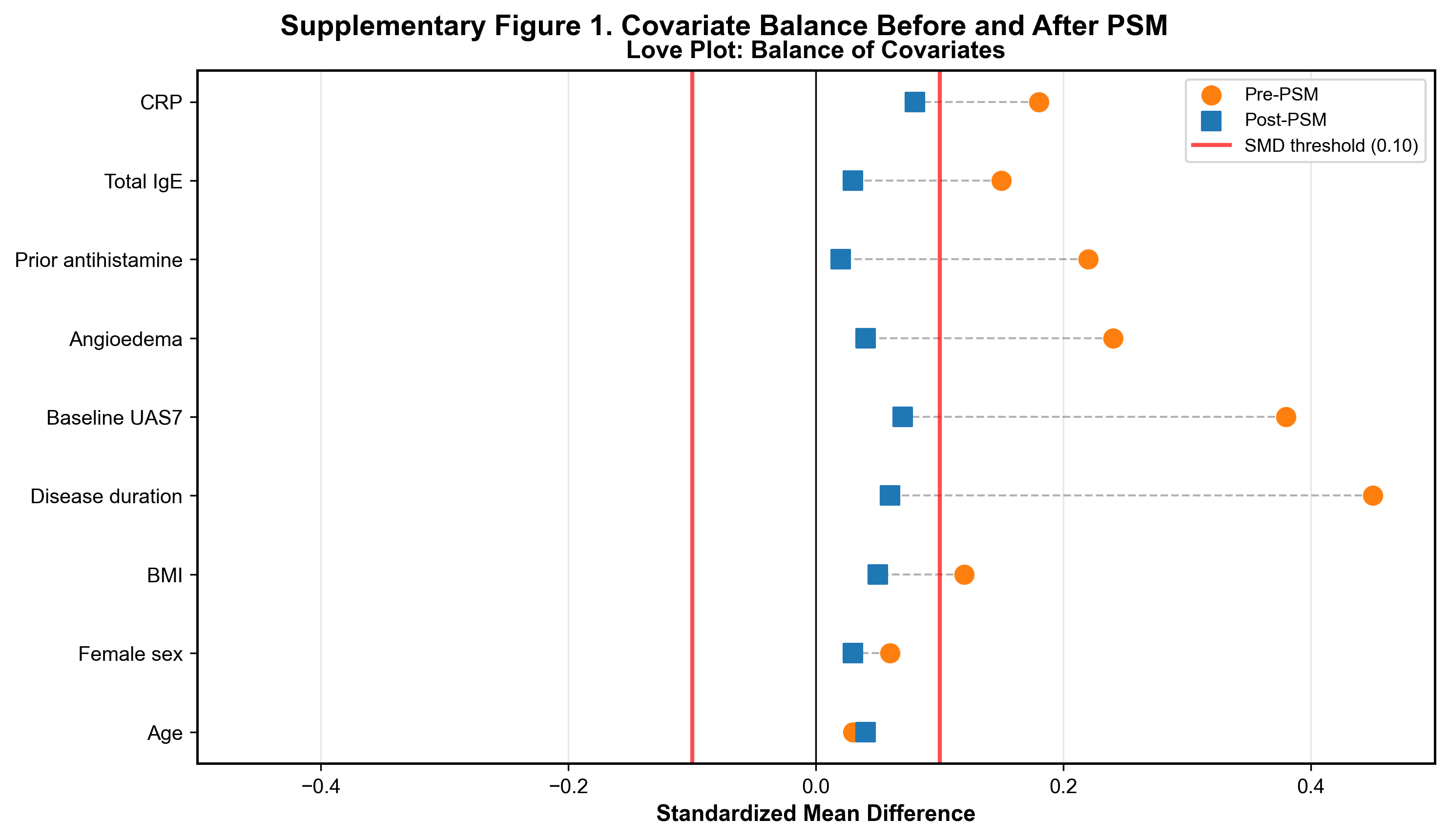


## ****Supplementary Figure 1. Covariate Balance Before and After PSM****

Love plot showing standardized mean differences for each covariate included in the propensity score model. Before matching, SMDs ranged from 0.02 to 0.45, exceeding the 0.10 threshold for disease duration, baseline UAS7, and CRP. After 1:1 nearest-neighbor matching, all SMDs were reduced to <0.08, demonstrating excellent covariate balance between antibody-positive and antibody-negative groups.


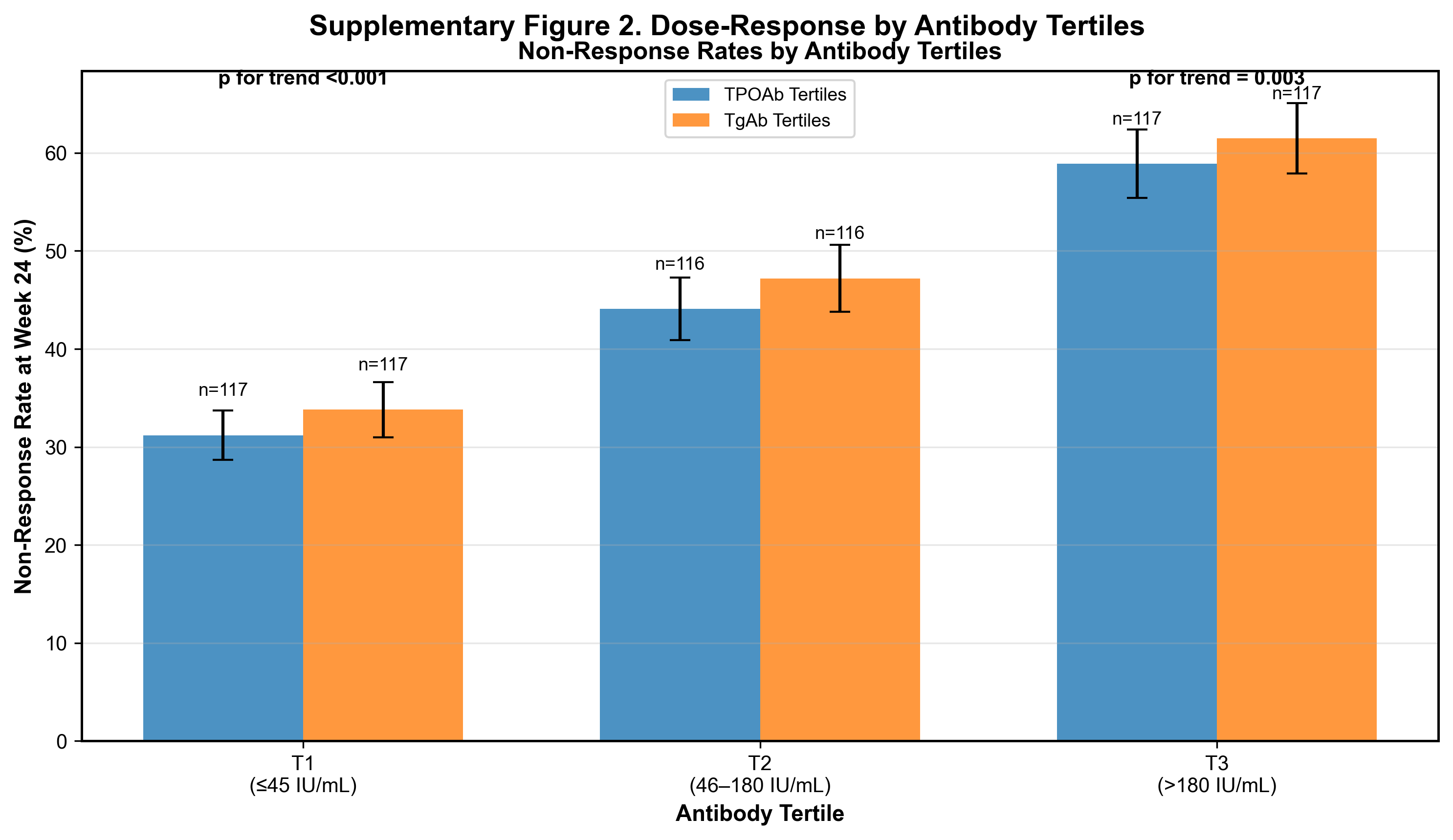


## ****Supplementary Figure 2. Dose-Response Relationship by Antibody Tertiles****

Bar graph displaying Week 24 non-response rates across TPOAb and TgAb tertiles in the overall cohort (n=350). TPOAb tertiles: T1 (0–45 IU/mL), T2 (46–180 IU/mL), T3 (>180 IU/mL). Non-response rates increased progressively: 31.2% in T1, 44.1% in T2, and 58.9% in T3 (p for trend <0.001). Similar pattern observed for TgAb tertiles (p for trend=0.003).
